# Supplementary material for: Matrigel 3D bioprinting of contractile human skeletal muscle models recapitulating exercise and pharmacological responses
Source: Commun Biol. 2021 Oct 14;4:1183. doi: 10.1038/s42003-021-02691-0 (PMC8516940; doi:10.1038/s42003-021-02691-0)
Supplement: Supplementary file 2 — Supplementary Information [file 42003_2021_2691_MOESM2_ESM.pdf]

Matrigel 3D bioprinting of contractile human skeletal muscle models recapitulating exercise and pharmacological responses

Angela Alave-Furrer[1], Sonia de Andrade[1,2], Dominic Bachmann[1], Heidi Jeker[1], Martin Steinmann[3], Nathalie Accart[1], Andrew Dunbar[3], Martin Rausch[3], Epifania Bono[2,4], Markus Rimann[2,4], Hansjoerg Keller[1]

[1]*Musculoskeletal Disease Area, Novartis Institutes for BioMedical Research, Basel, Switzerland;* [2]*3D Tissues and Biofabrication, Institute of Chemistry and Biotechnology (ICBT), Zurich University of Applied Sciences, Waedenswil, Switzerland;* [3]*Analytical Sciences and Imaging, Novartis Institutes for BioMedical Research, Basel, Switzerland;* [4]*Competence Center TEDD, Institute of Chemistry and Biotechnology (ICBT), Zurich University of Applied Sciences, Waedenswil, Switzerland.*

Supplementary Information

Supplementary Figure 1. Microvalve drop formation and impurities. a) Images of drop formation at the printhead microvalve orifice during printing over time as indicated in the figure. For better visualization, the drop is highlighted with a white dotted curve. **b)** Image of a clean microvalve orifice. **c)** & **d)**, Images of impurities in two used microvalves beneath the orifice.

a)
b)
c)
d)

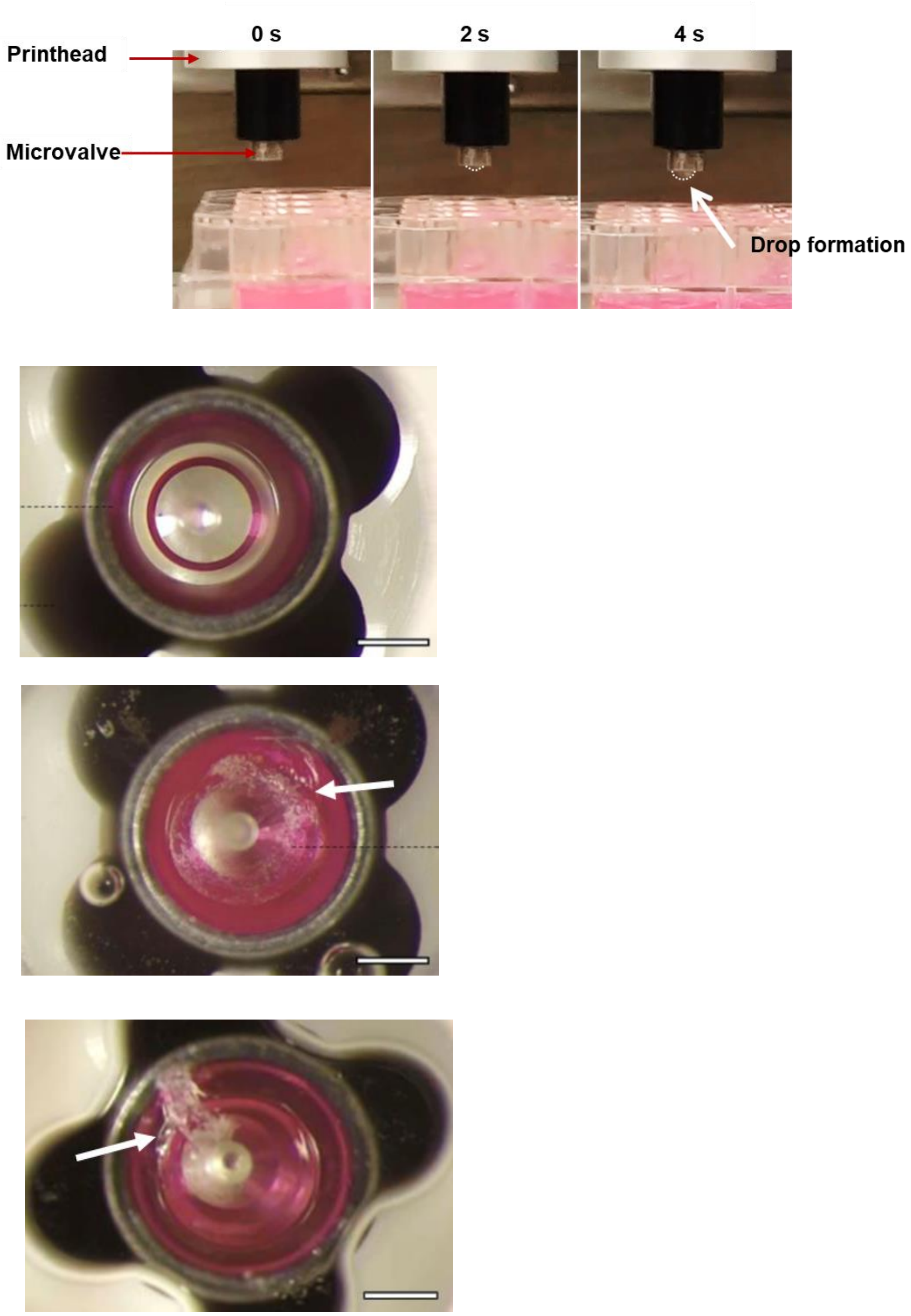

Supplementary Figure 2. Custom-made electrical circuit board with electrodes for 24-well plate EPS. a) Top view of the custom-made electrical circuit board for 24-well plate EPS. A rectangular window for each well allows optical inspection of the muscle models with a bin-ocular. The ribbon cable connection of the board to the control system is seen on the right. **b)** Coverage of the circuit board with a 24-well plate lid for long term incubation. **c)** Bottom view of the circuit board with inserted U-shaped Pt electrodes.

a)

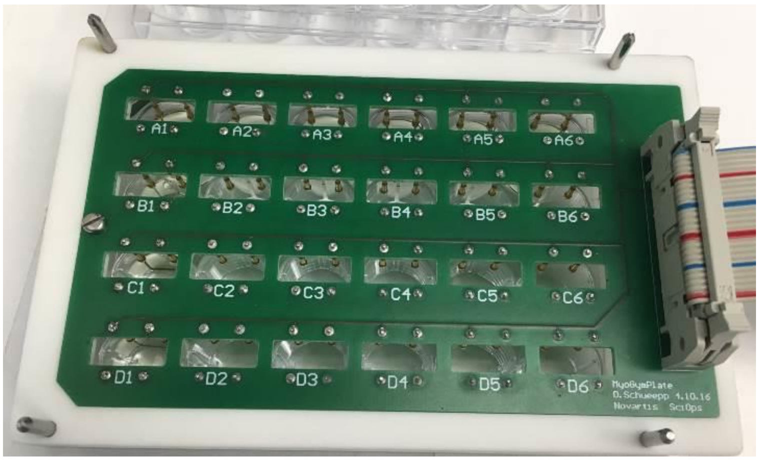

b)

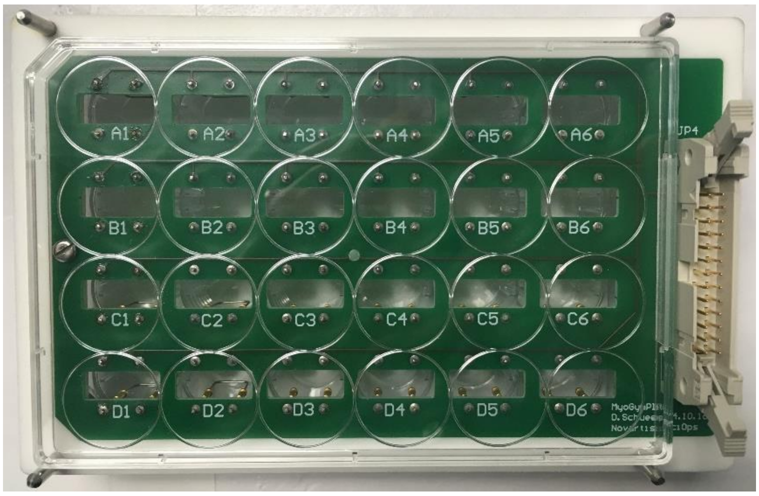

c)

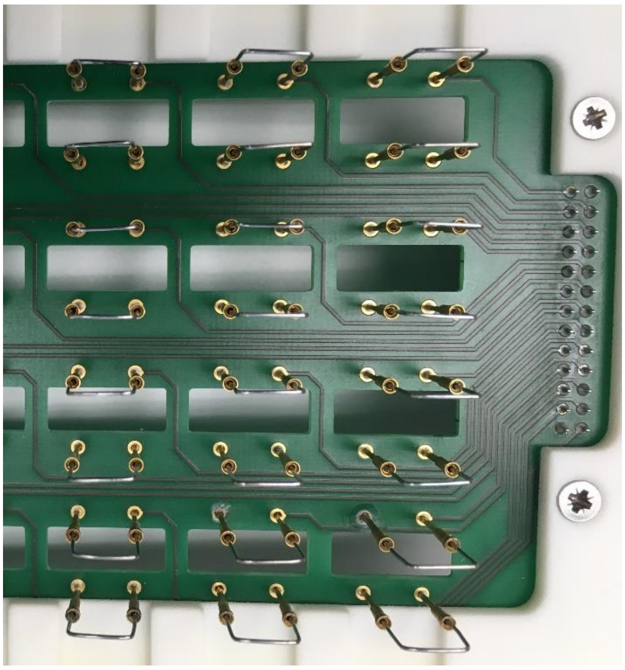

Supplementary Table 1. List of TaqMan assays

Gene	TaqMan assay
18S	4319413E
ACTN2	Hs00153809_m1
B2M	Hs00187842_m1
DES	Hs00157258_m1
GAPDH	43108843E
IL6	Hs00174131_m1
MYF5	Hs00271574_m1
MYH1	Hs00428600_m1
MYH2	Hs00430042_m1
MYH3	Hs01074230_m1
MYH7	Hs01110632_m1
MYH8	Hs00267293_m1
MYOD1	Hs00159528_m1
MYOG	Hs01072232_m1
TBP	4326322E
