# Supplementary material for: Matrigel 3D bioprinting of contractile human skeletal muscle models recapitulating exercise and pharmacological responses
Source: Commun Biol. 2021 Oct 14;4:1183. doi: 10.1038/s42003-021-02691-0 (PMC8516940; doi:10.1038/s42003-021-02691-0)
Supplement: Supplementary file 3 — Description of Additional Supplementary Files [file 42003_2021_2691_MOESM3_ESM.pdf]

Description of Additional Supplementary Files

File name: Supplementary Movie 1.
Description: Recording of EPS-induced (1 ms pulse, 25 Hz, 300 ms, 400 mA) model contractions in a representative model from a 17-years old donor differentiated for 17 days.

File name: Supplementary Movie 2.
Description: Recording of a littermate control model activity after the addition of 10 µg/mL myosin inhibitor blebbistatin.

File name: Supplementary Data 1.
Description: All source data underlying graphs.
